# Supplementary material for: Sociodemographics and health-literacy as predictors of cervical cancer screening practices among Haitian women: A secondary data analysis of 2016–17 DHS surveys
Source: PLOS Glob Public Health. 2023 Aug 8;3(8):e0002221. doi: 10.1371/journal.pgph.0002221 (PMC10409270; doi:10.1371/journal.pgph.0002221)
Supplement: Attachment — Submitted filename: Reviewer Response.docx [file pgph.0002221.s001.docx]

| Reviewer Comments | Author Response |
| --- | --- |
| Editor Comments |  |
| Please review your reference list to ensure that it is complete and correct. If you have cited papers that have been retracted, please include the rationale for doing so in the manuscript text, or remove these references and replace them with relevant current references. Any changes to the reference list should be mentioned in the rebuttal letter that accompanies your revised manuscript. If you need to cite a retracted article, indicate the article’s retracted status in the References list and also include a citation and full reference for the retraction notice. | Our reference list has been revised to ensure there are no retracted papers cited. |
| There is need to revise the title of the study to read; "Sociodemographics and Health-literacy as predictors of cervical cancer screening Practices among Haitian women: A secondary data analysis of 2016-17 DHS surveys". The rationale is that the use of Health-literacy best contextualize the argument of the study as mentioned in the discussion and many instances literacy and information sources in the context understanding the issues around cancer of the cervix are modifiable risk factors that can be ameliorated by health education through the identified information media. | Edits have been made to the Title per the suggestions provided as the authors agree that this change is a much stronger reflection of the aims of this article. |
| ABSTRACT: (1) The media through which the at-risk population may access health-related information may be regarded as important resources to improve health literacy. Therefore revise the objective to align with health-literacy. Kindly note that the conclusion also justifies the basis of replacing literacy and sources of information with health-Literacy as a construct in the study. | We have revised the objectives under the Abstract to reflect sources of health information and health literacy as being determinants of engagement in cervical cancer screening among women in Haiti. |
| INTRODUCTION: In the introduction, the second sentence should read: "Haiti reports the highest rates of cervical cancer incidence and mortality…" | Thank you for this suggestion, we have made edits to the second sentence of the introduction per the recommendation provided. |
| Second paragraph argues of favorable preventable outcomes related to public health principles of prevention and control but fail to adequately review the scientific context necessary to elucidate the dynamics of the observed poor screening uptake the basis of which constitute the modifiable risk factors the variables in the study are predicated. It would have been very appropriate to review the theoretical clarifications needed to understand the dynamics attempted in the third paragraph: "In the context of cancer screening, a growing body….may contribute to cervical cancer risk among Haitian women" | Further information has been provided on the modifiable factors associated with uptake of cervical cancer screening with a focus on the lack of literature exploring the role of health literacy and sources of health information in cervical cancer screening within LMICs. We state that health literacy and sources of information may contribute towards individuals’ cervical cancer and cervical cancer screening knowledge levels, perceived susceptibility, perceived benefits and barriers, and perceived self-efficacy in engagement in cervical cancer screening; thus informing perceptions and attitudes towards engagement in cancer screening. |
| The omission of an articulated underpinning theoretical foundation involving the modifiable risk factors in this study representing the framework constitute a serious omissions that invalidates any attempt to elucidate the dynamics of the observed outcomes of poor screening practices. The argument appears superficial and not grounded in health promotion theories, though mention is made about "primary prevention", "secondary prevention" "utilization of screening" these are not linked with the source theories and cannot provide the needed proof of concept the objective of the study seeks to elucidate. | We have now included a statement in the Introduction section regarding how health literacy and sources of health information can potentially influence the Health Belief Model Constructs of perceived susceptibility, perceived benefits and barriers, perceived self efficacy, and perceived severity which can have an impact on an individual’s decision to undergo cervical cancer screening. |
| The mention of "preventive health behaviour" and all the constructs within the context of health-seeking governing preventive health explainable by the health belief model demands the review of this foundational framework before the statement of purpose "...to assess determinants of engagement in cervical cancer screening among…". | As mentioned in the prior comments, we have included a statement on the role of health literacy and sources of information on the varying HBM constructs. In addition, we have provided examples of studies which have showcased that low levels of cancer literacy can contribute to low uptake of screening. |
| RESULTS:(Subsection reporting Literacy and Sources of Information) There is a need to revise the statement in the first sentence: "...sample had nearly equal amounts of women who..." to "...sample had nearly equal numbers of women who..." In the results how did they compare with screening outcomes? | We have revised the first sentence under ‘Literacy and Sources of Information’ per the recommendations provided.  We have included information on screening outcomes between literate and illiterate groups per our Chi2 analysis. Among those who were literate, 10.75% (n=33) had been screened. Among those who were not literate , less than 5% (3.96%, n=12) had received screening. |
| DISCUSSION: In initiating the discussion for this study, it would have been very appropriate to reiterate the objectives of the study to maintain alignment with the thesis of the study. The thesis of the study includes emerging questions warranting the study needing answers such as why is cervical cancer highest in Haiti, why are Haitian women not engaged in cervical screening practices for likely early detection or even preventive vaccination practices among others? Then proceed to review finding and explain likely reasons for what the data has revealed. The would strengthen such a fine scientific work. | The authors have made edits to reiterate the objectives of the study in the first paragraph of the discussion. We provide a statement indicating structural and individual barriers that may be contributors to low uptake of cervical cancer screening in Haiti, however we have expanded on this topic significantly in the Introduction and thus do not provide detail in the Discussion to avoid being repetitive. In the Introduction, we have also previously stated that HPV vaccines are not available to the public sector in Haiti, thus vaccination is not a primary means of cervical cancer prevention. |
| The second paragraph begins with a statement; "...evaluated the influence of sociodemographics on receiving cervical cancer screening." It would be most appropriate to use "associated with" rather than "influence" this study cannot demonstrate influence but association or demonstrate predictive value of variables. Only experimental studies can infer impact, influence or effect. | Thank you for this feedback, we have made edits to rephrase the statement in the second paragraph per the recommendations provided. |
| Were there measures of engagement in screening in the study? | Engagement in cervical cancer screening was measured as a binary ‘Yes or No’ variable which we have detailed under our ‘Methods’ section. |
| Reviewer 1 Comments |  |
| The authors need to provide the following data on Age, Religion, Marital Status and Employment Status and insert into Table 2. This is because the authors have mentioned that these socio-demographic data were part of what was analysed. These variables are missing in the Table 2. Hence it will be wrong to deduce that these are statistically significant with the odds of undergoing cervical screening. | In our analysis we did a univariate regression followedby a multivariate regression. Significant variables from the univariate model were subsequently inserted into the multivariate regression model. We have described this under our Methods, “STATA software was used for the analysis(35). Descriptive statistics were used to describe the sample. Univariate logistic regression was conducted to evaluate the significance of covariates on engagement in cervical cancer screening. Variables that demonstrated significance in the univariate regression were included in the multivariate logistic regression models. Two multivariate models were developed, the first evaluated the influence of sociodemographics on receiving cervical cancer screening. Ghe second model evaluated the influence of literacy and sources of information on receiving cervical cancer screening. In all analyses, statistical significance was set at p≤0.05.”  Age, Religion, Marital Status, and Employment Status covariates did not demonstrate significance in the univariate model and thus were not incorporated into the multivariate model. Because these covariates did not demonstrate significance in either models, they were not included in Table 2. |
| Studies have reported that these variables Age, Religion, Marital Status and Employment Status are significant to determine whether or not women will undergo cervical screening. See the following Articles Below as a reference. • e study. | The recommended articles to cite have not been provided in the reviewer response. However, we have provided citations to substantiate the claim that sociodemographics have demonstrated significance in cervical cancer screening within previous studies conducted amongst Haitian populations-   1. McCarthy SH, Walmer KA, Boggan JC, Gichane MW, Calo WA, Beauvais HA, et al. Awareness of Cervical Cancer Causes and Predeterminants of Likelihood to Screen among Women in Haiti. J Low Genit Tract Dis [Internet]. 2017 Jan 1 [cited 2021 Apr 22];21(1):37–41. Available from: /pmc/articles/PMC5545975/ 2. Guillaume D, Amedee LM, Rolland C, Duroseau B, Alexander K. Exploring engagement in cervical cancer prevention services among Haitian women in Haiti and in the United States: A scoping review. J Psychosoc Oncol. 2022 3. Menard J, Kobetz E, Maldonado JC, Barton B, Blanco J, Diem J. Barriers to Cervical Cancer Screening Among Haitian Immigrant Women in Little Haiti, Miami. J Cancer Educ 2010 254 [Internet]. 2010 Mar 16 [cited 2021 Oct 2];25(4):602–8. Available from: https://link-springer-com.proxy1.library.jhu.edu/article/10.1007/s13187-010-0089-7 |
| Correct “Ghe” to “The” on Line 7 under Data Analysis section of the Paper. | Thank you for noting this error, we have made edits for clarity. |
| The authors should incorporate the word “variables” to buttress their explanation of the socio-demographic data set. | We have incorporated the term ‘variables’ as recommended under ‘Sociodemographics.’ |
| Reviewer 2 Comments |  |
| The manuscript is technically sound, and the data supports the conclusions. The manuscript describes methodologically and ethically rigorous research with conclusions that are appropriately drawn based on the data presented. | Thank you for taking the time to review our manuscript and provide feedback. |
| The authors did not make all data underlying the findings in their manuscript fully available. This is most likely due to the fact that third party data - from the Haitian USAID Demographic and Health Surveys (DHS) - was analyzed in the study. | The USAID dataset is a publicy available dataset which we have mentioned within our manuscript. The data is fully available to researchers who register to access their database, and we have provided a citation for individuals to access the database. Thus, we did not make the dataset available within this manuscript. |
| The language of the manuscript is clear, correct, and unambiguous. However some typographical and grammatical errors were identified in the attached manuscript. | We have reviewed our manuscript for grammatical errors and have made changes accordingly. |
